# Selective Depletion of Regulatory T Cell Subsets by Docetaxel Treatment in Patients with Nonsmall Cell Lung Cancer

**DOI:** 10.1155/2014/286170

**Published:** 2014-04-28

**Authors:** Jie-Yao Li, Xiu-Fang Duan, Li-Ping Wang, Yu-Jie Xu, Lan Huang, Teng-Fei Zhang, Jin-Yan Liu, Feng Li, Zhen Zhang, Dong-Li Yue, Fei Wang, Bin Zhang, Yi Zhang

**Affiliations:** ^1^Department of Oncology, The First Affiliated Hospital of Zhengzhou University, Zhengzhou, Henan 450052, China; ^2^Biotherapy Center, The First Affiliated Hospital of Zhengzhou University, Zhengzhou, Henan 450052, China; ^3^School of Life Science, Zhengzhou University, Zhengzhou, Henan 450001, China; ^4^Department of Medicine-Division of Hematology/Oncology, Robert H. Lurie Comprehensive Cancer Center, Northwestern University Feinberg School of Medicine, Chicago, IL 60611, USA; ^5^Key Laboratory of Clinical-Medicine, The First Affiliated Hospital of Zhengzhou University, Zhengzhou, Henan 450052, China

## Abstract

Regulatory T (Treg) cells are potent suppressors that maintain immune homeostasis. Accumulation of Treg can inhibit effective immune responses in cancer patients, leading to tumor development and progression. Despite direct cytotoxicity, several chemotherapeutic drugs have been reported to deplete Treg cells for better prognosis for cancer patients. Treg cells are a heterogenous population with at least three different subsets, nonsuppressive, resting, and activated Treg cells. However, the characteristics of Treg cell subsets in lung cancer patients and how chemotherapy affects Treg cells remain elusive. In this study, we first analyzed Treg cell subsets in peripheral blood samples from 40 nonsmall cell lung cancer (NSCLC) patients and 20 healthy donors. Treg cells, specifically activated Treg cell subset, significantly increased in patients with NSCLC. Compared to nonsuppressive Treg cells, activated Treg cells expressed higher level of CD39 and predominantly produced inhibitory cytokines. *In vitro* assay showed that docetaxel reduced all three subsets of Treg cells. More importantly, we found docetaxel-based chemotherapy significantly decreased all three Treg subsets after 4 cycles of treatment in 17 NSCLC patients. Taken together, this study revealed dynamic changes of various Treg cell subsets in NSCLC patients before and after chemotherapy, providing activated Treg cells as a potential target for chemotherapy.

## 1. Introduction

Regulatory T cells (Treg cells) are a key member to maintain self-tolerance and immune homeostasis [1, 2]. They play crucial roles in a variety of human diseases, such as autoimmune disease, allergy, chronic infection, and cancers [3–6]. Treg cells can suppress the immune response of CD4^+^ and CD8^+^ T cells mainly by secretion of inhibitory cytokines such as interleukin (IL)-10 and transforming growth factor-*β* (TGF-*β*) [7, 8]. Foxp3 is the most specific marker for CD4^+^ Treg cell development and function [9–11]. Sakaguchi's group confirmed that human CD4^+^Foxp3^+^ Treg cells can be divided into three subsets: CD45RA^+^Foxp3^lo^, CD45RA^−^Foxp3^hi^, and CD45RA^−^Foxp3^lo^ cells [12]. CD4^+^CD45RA^+^Foxp3^lo^ Treg cells as antigen-experienced cells are referred to as resting Treg cells (rTreg) [13–15]. CD4^+^CD45RA^−^Foxp3^hi^ Treg cells which are activated with highly suppressive function and proliferating ability* in vivo* are defined as activated Treg cells (aTreg) [13–15]. CD4^+^CD45RA^−^Foxp3^lo^ Treg cells include a remarkable amount of nonregulatory, cytokine-secreting T cells (nonsuppressive T cells or non-Treg cells) [13–15].

Elevated Treg cells reduce immune responses against tumor and induce excessive tumor progression [16, 17]. CD4^+^CD25^+^ Treg cells are augmented in tumor tissue as well as in circulation in patients with malignant melanoma, Hodgkin lymphoma, and lung, gastric, ovarian, pancreatic, and breast cancer [18–20]. Traditionally, the aim of chemotherapy is direct cytotoxicity to induce tumor cell death. Taxanes containing docetaxel or paclitaxel have been used to treat a variety of malignancies such as lung, prostate, and breast cancers. They have also been reported to modulate components of the immune system in mice by disrupting intracellular microtubular networks [21]. In those studies, docetaxel showed clear antitumor effects and further enhanced antitumor effects by modulation of immune cell subsets or regulatory T cells. However, no study has demonstrated the effect of docetaxel on the frequency and function of individual Treg cell subsets.

In this report, we characterized three subsets of CD4^+^Foxp3^+^ Treg cells in NSCLC patients. Only aTreg cells have been found to increase in NSCLC patients, especially in patients with advanced NSCLC. We also identified the relationship between three Treg subsets and pathological characteristics. Finally, our data demonstrate that docetaxel modulates different subsets of Treg cells both in* in vitro* analysis and* in vivo* clinical settings.

## 2. Materials and Methods

### 2.1. Patients and Blood Samples

From February 2013 to November 2013, 40 patients with NSCLC from the First Affiliated Hospital of Zhengzhou University were enrolled. The patients have not been treated with anticancer drugs, radiotherapy, or surgery in the beginning of the study and have no other systemic diseases. Peripheral blood was collected from 40 patients with NSCLC and 20 healthy donors with similar gender and age distribution, respectively. All patients gave written informed consent. The whole consent procedure was in accordance with the standard defined by Institutional Review Boards of the First Affiliated Hospital of Zhengzhou University. Patient characteristics were summarized in Table 1.

### 2.2. Antibodies and FACS Analysis

Fresh human peripheral blood mononuclear cells (PBMCs) were stained with anti-CD4 (PerCP-Cy 5.5 or APC-Cy7-conjugated from BD Bioscience), anti-CD25 (APC-Cy7 or APC-conjugated from BD Bioscience), and anti-CD45RA (FITC-conjugated from BD Bioscience). Intracellular detection of Foxp3 with anti-Foxp3 (PE-conjugated from BD Bioscience) was performed on fixed and permeabilized cells with the Foxp3 staining buffer set (Biolegend, USA) according to the manufacturer's instructions. The following fluorescence-conjugated antibodies were also used: CD39 (APC), Interferon-*γ* (IFN-*γ*) (PE-Cy7 or APC), and TGF-*β* (APC) obtained from BD Biosciences. PBMCs were stained according to the manufacturer's recommendations. The appropriate isotype-matched control antibodies were purchased from BD Bioscience. Cells were analyzed using a FACSCantoII flow cytometer (BD, USA) and Diva analysis software (BD, USA).

### 2.3. Intracellular Staining

Intracellular staining for IFN-*γ* and TGF-*β* was performed as follows: PBMCs were freshly isolated and stimulated with 1 mg/mL PMA (Sigma, USA) and 1 mg/mL ionomycin (Sigma, USA) in the presence of Brefeldin-A (BFA, Biolegend, USA) for 5 h. Cells were stained for cell surface markers and then fixed and permeabilized with anti-human Foxp3 Ab for intracellular cytokine staining. FACSCanto II flow cytometer (BD, USA) was used to determine fluorescence intensity and Diva analysis software was used to analyze the data.

### 2.4. Cell Isolation and Sorting

PBMCs were isolated by density gradient centrifugation (Tianjin HY, China) within 2 h after sample collection. There is a linear correlation between CD25 and Foxp3 levels expressed on CD4^+^CD25^+^ T cells [7]. To isolate live Treg subsets for functional assays, the PBMCs were stained with CD4 and CD25 Abs and sorted using Moflo-XDP (Beckman Coulter, USA) according to the manufacturer's instructions. The purity of CD4^+^CD25^+^ T cells was >90%, confirmed by flow cytometry (data not shown).

### 2.5. *In Vitro* Assay of Docetaxel Effect on the Treg Subsets

The purified CD4^+^CD25^+^ T cells were resuspended in RPMI1640 (Gibco, USA) containing 10% fetal bovine serum (FBS, Sigma, USA), 100 U/mL penicillin, and 100 *μ*g/mL streptomycin. After 24 h of incubation in the atmosphere with 5% CO_2_ at 37°C, 100 IU/mL IL-2 (Beijing SL, China) and 1 *μ*g/mL docetaxel (Zhejiang WM, China) were added. Assay cultures after 36 h were harvested and ready for phenotype and cytokine analysis of the three subsets of Tregs, aTreg, rTreg, and non-Treg cells being, respectively, defined as CD4^+^CD45RA^−^CD25^hi^, CD4^+^CD45RA^+^CD25^lo^, and CD4^+^CD45RA^−^CD25^lo^ T cells.

### 2.6. Therapeutic Regimen

Of these 40 NSCLC patients, 17 received cisplatin (75 mg/m^2^) plus docetaxel (30 mg/m^2^ on day 1 and day 8) every three weeks. All patients were treated for 4 cycles.

### 2.7. Statistical Analysis

Differences between groups were assessed using Student's *t*-test and paired *t*-test. The correlation between Treg cell subsets and clinical characters was determined by one-way ANOVA. The change of Treg cells treated with docetaxel was determined by randomized block design ANOVA. *P* values were considered significant at *P* < 0.05 (*P < 0.05; **P < 0.01; ***P < 0.001). Statistical analyses were performed in SPSS version 17.0.

## 3. Results

### 3.1. Only aTreg Cells Increased in NSCLC Patients

**Figure 1:**
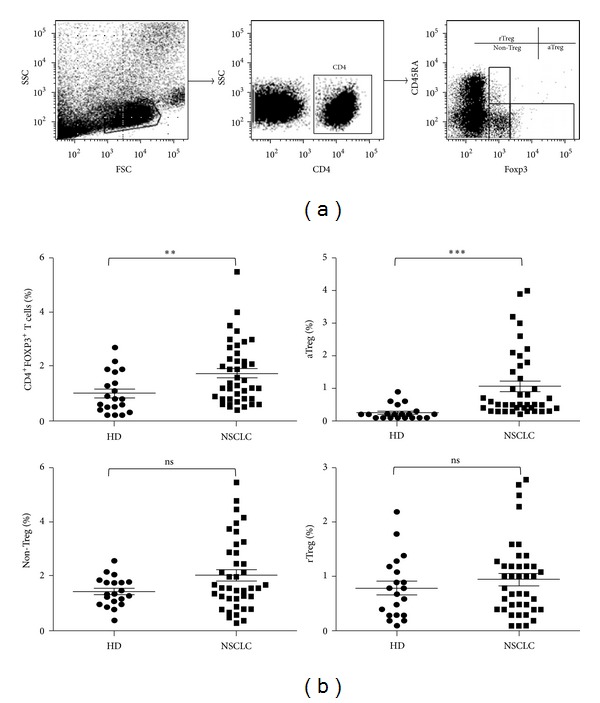
aTreg but not rTreg and non-Treg cells increased in NSCLC patients. (a) CD4^+^Foxp3^+^ T cells and three subsets of Treg cells from PBMCs were isolated and analyzed by FACS. (b) The percentages of Treg cells (CD4^+^Foxp3^+^ T (total Treg) cells, CD4^+^CD45RA^−^Foxp3^hi^ (aTreg) cells, CD4^+^CD45RA^−^Foxp3^lo^ (non-Treg) cells, and CD4^+^CD45RA^+^Foxp3^lo^ (rTreg) cells) in CD4^+^ T cells were calculated after FACS analysis. HD: healthy donor, *n* = 20; NSCLC: nonsmall cell lung cancer, *n* = 40. Each dot represents one individual sample. **P < 0.01 and ***P < 0.001 for statistical analysis by Student's *t*-test.

The combination of Foxp3 and CD45RA staining of CD4^+^ T cells in PBMCs of NSCLC patients revealed the existence of three subsets of Treg cells (Figure 1(a)). Notably, these three CD4^+^Foxp3^+^ populations could be distinctly separated into Foxp3^lo^CD45RA^+^ cells (rTreg cells), Foxp3^hi^CD45RA^−^ cells (aTreg cells), and Foxp3^lo^CD45RA^−^ cells (non-Treg cells). As shown in Figure 1(b), the percentage of CD4^+^Foxp3^+^ Treg cells from PBMCs increased in NSCLC patients compared to healthy donors (1.76 ± 0.17% versus 1.01 ± 0.16%, *P* < 0.01). We further analyzed three subsets of CD4^+^Foxp3^+^ cells in total CD4^+^ T cells. Our data showed that only aTreg cells but not rTreg or non-Treg cells increased in NSCLC patients compared to healthy donors (1.07 ± 0.16% versus 0.25 ± 0.04%, *P* < 0.001), indicating that aTreg cells might play an important role in the pathogenesis of lung cancer.

### 3.2. Activated Treg Cells Expressed Higher Levels of CD39 and Inhibitory Cytokines in Patients with NSCLC

**Figure 2:**
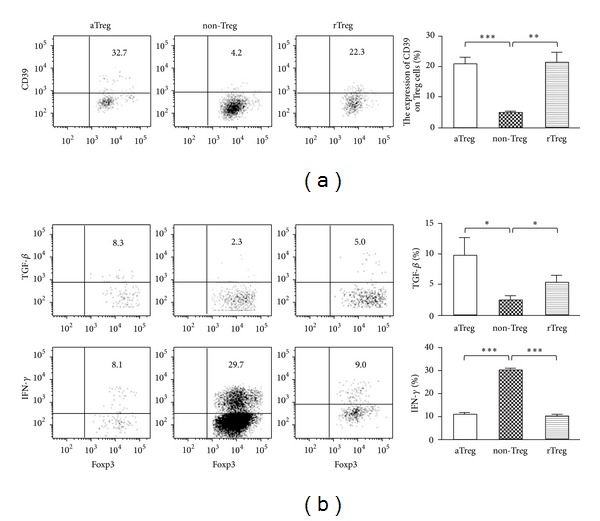
aTreg cells expressed higher immunosuppressive marker CD39 in NSCLC patients and secreted suppressive cytokines. (a) PBMCs were collected from NSCLC patients. The phenotype marker of CD39 was evaluated in the three subsets of CD4^+^Foxp3^+^ Treg cells, including aTreg, non-Treg, and rTreg cells. The dot plots (left) represent the expression of CD39 in each group. The bar figures (right) represent the mean percentage of each population ± standard error of mean. (b) These cells were also stimulated* in vitro* and the cytokine profiles including TGF-*β* and IFN-*γ* were analyzed. *P < 0.05; **P < 0.01; ***P < 0.001 by paired *t*-test.

To evaluate the suppressive function of Treg subsets, we further detected the phenotypes of different Treg subsets in patients with NSCLC. CD39 is an ectonucleotidase and has been defined as an additional important marker for Treg cells, which converts extracellular ATP into immunosuppressive adenosine [22]. CD39 has been defined as an additional important functional marker for Treg cells [23]. So, CD39 expression was detected in the three Treg cell subsets. We found that CD39 was enriched in aTreg and rTreg cells in comparison to non-Treg cells in PBMCs (Figure 2(a)). We also studied the cytokine pattern in Treg cell subsets in PBMCs from NSCLC patients after* ex vivo *stimulation. As shown in Figure 2(b), aTreg cells secreted significant amount of TGF-*β* (*P* < 0.05) but very little INF-*γ* (*P* < 0.001) compared to non-Treg cells. In contrast, non-Treg cells predominantly secreted INF-*γ* but not TGF-*β*. Activated Treg cells also secreted more TGF-*β* than rTreg cells, but the difference is not significant. These characteristics suggest that aTreg cells were the major Treg subset with inhibitory function in NSCLC patients.

### 3.3. Activated Treg Cells Correlated with Advanced Pathological Stages in NSCLC Patients

**Figure 3:**
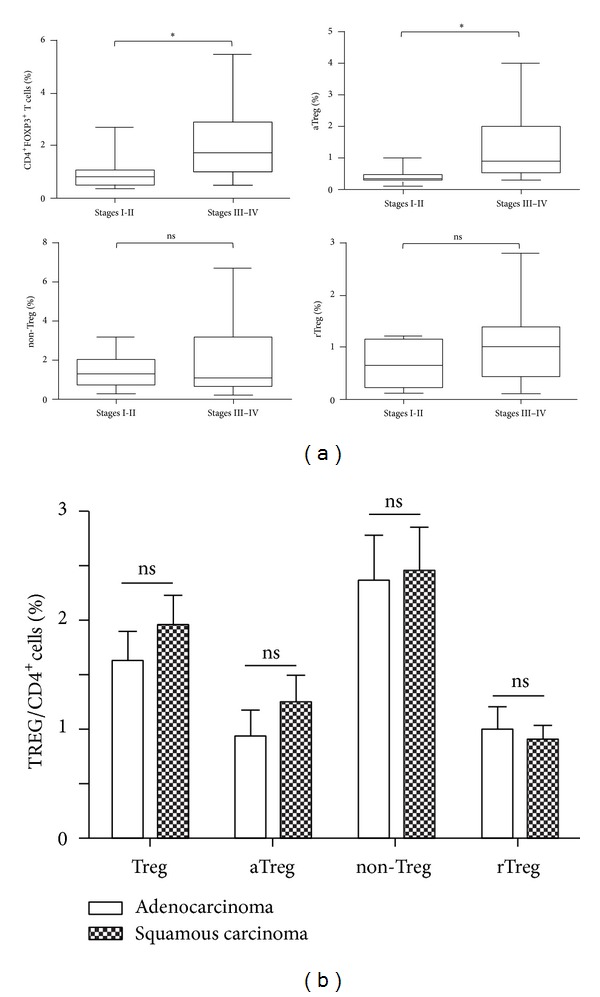
aTreg cells had a higher level in patients with advanced NSCLC. NSCLC patients were grouped according to clinical stage and pathology. (a) The percentages of CD4^+^Foxp3^+^ T cells, aTreg cells, non-Treg cells, and rTreg cells were compared in PBMCs of NSCLC patients at stages I-II and III-IV. (b) The 4 groups of Treg cells were compared in PBMCs of NSCLC patients between adenocarcinoma and squamous carcinoma. Statistical analysis was determined by one-way ANOVA. *P < 0.05.

The clinical relevance of Treg cell subsets with tumor stages and other pathological factors was examined. In thirty-two NSCLC patients at stages III-IV, the percentage of CD4^+^Foxp3^+^ Treg cells in PBMCs was significantly higher than that in patients at stages I-II (2.01 ± 0.23% versus 0.98 ± 0.25%, *P* < 0.05, Figure 3(a)). We also evaluated whether the subsets of Treg cells correlated with tumor stages. The frequency of aTreg cells was much higher in patients with NSCLC at stages III-IV (1.30 ± 0.18% versus 0.38 ± 0.09%, *P* < 0.05, Figure 3(a)). However, there were no significant differences in the subsets of Treg cells between different types of histology (Figure 3(b)).

### 3.4. Effect of Docetaxel on Three Subsets of Treg Cells

**Figure 4:**
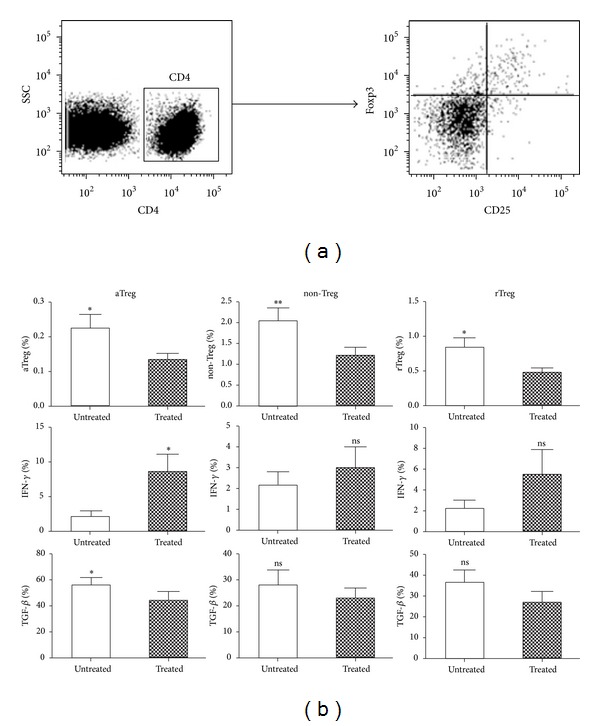
The three Treg subsets reduced after being treated with docetaxel* in vitro*. (a) PBMCs from NSCLC patients were staining with CD25 and Foxp3 antibody and analyzed by flow cytometry. (b) The peripheral blood of NSCLC patients was collected and CD4^+^CD25^+^ T cells were isolated by flow cytometric sorting. Three Treg subsets were defined with CD4^+^CD45RA^−^CD25^hi^ (aTreg) cells, CD4^+^CD45RA^−^CD25^lo^ (non-Treg) cells, and CD4^+^CD45RA^+^CD25^lo^ (rTreg) cells. The differences of three subsets with or without docetaxel were analyzed. *P < 0.05; **P < 0.01 by paired *t*-test.

Previous studies have shown that docetaxel induced tumor cell death and also increased the number of CD4^+^ and CD8^+^ T cells [24]. We investigated if docetaxel had different effects on each Treg subset. To address this issue, we treated purified CD4^+^CD25^+^ T cells derived PBMCs from NSCLC patients with docetaxel* in vitro*. Because the degree of Foxp3 was proportional to CD25 expression (Figure 4(a)), we isolated and defined aTreg, rTreg, and non-Treg cells as CD4^+^CD45RA^−^CD25^hi^, CD4^+^CD45RA^+^CD25^lo^, and CD4^+^CD45RA^−^CD25^lo^ T cells. Three subsets of Treg cells were all decreased after docetaxel treatment. More interestingly, aTreg cells secreted more INF-*γ* and less TGF-*β* after docetaxel treatment (*P* < 0.05, Figure 4(b)). But there were no significant differences for cytokine production in rTreg and non-Treg cells after docetaxel treatment.

**Figure 5:**
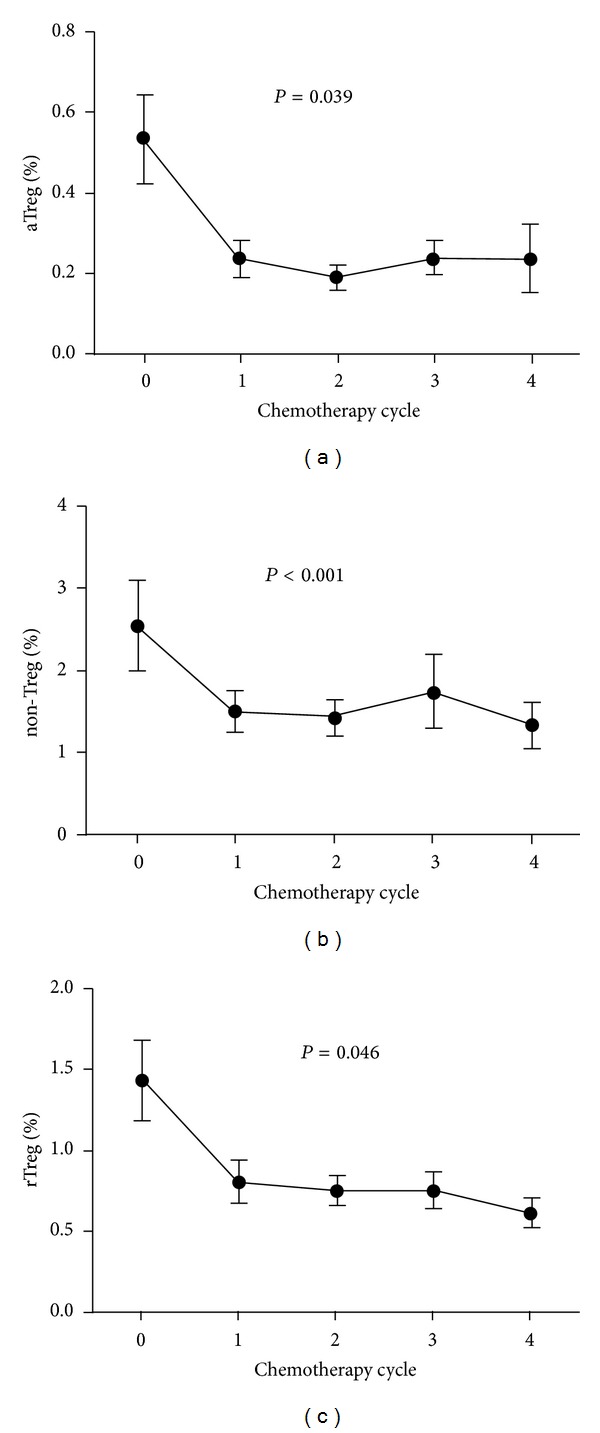
Treg cell subsets of NSCLC patients were decreased after chemotherapy. The patients with NSCLC received 4 cycles of chemotherapy and the peripheral blood was collected 1 day before the first cycle and 2 weeks after each cycle. Three subsets were analyzed by FACS. Statistical analysis was determined by randomized block design ANOVA. *P < 0.05.

To further confirm the clinical effect of docetaxel on Treg subsets, we collected peripheral blood from NSCLC patients 1 day before the first cycle and 2 weeks after each cycle of docetaxel treatment. As shown in Figure 5, three subsets of Treg cells were reduced after four cycles of chemotherapy (*P* < 0.05). The trend we observed coincided with the results observed* in vitro*.

## 4. Discussion

The adaptive immune system plays an important role in control of tumor development. Treg cells increased in most human solid tumors and can suppress antitumor immune responses by inhibition of tumor-specific CD8 T cells [24]. More and more reports showed that the increased number of Treg cells in solid tumors was related to greater tumor progress and poorer survivals [25]. Recently, CD4^+^Foxp3^+^ Tregs in tumor tissue were reported to have significantly increased compared with normal lung tissue [26]. In this study we reported significant increase of aTreg cells in peripheral blood of NSCLC patients. Consistent with previous studies, we confirmed that Treg cells increased in PBMCs. Furthermore, our results characterized three distinct subsets of Treg cells in NSCLC patients and revealed the relationship between Treg subsets and several pathological factors. The conversion of non-Treg cells to Treg cells is one of the mechanisms to promote the accumulation of Treg cells in suppressing antitumor immune response [27]. Activated Treg cells are highly proliferative* in vivo *but rapidly died, and rTreg cells can differentiate to aTreg cells under stimulation. However, we did not detect the translation of rTreg in NSCLS. Our results showed that depleting Treg cells might be therapeutically beneficial for the tumor immunotherapy [13].

Multiple mechanisms have been reported for Treg cells implicating in the immune suppression of human cancer, which may be potential target for depleting Tregs for immunotherapy [28]. In order to explore the potential factors contributing to the conversion in Treg cells, we identified the differences of cytokines and other cell surface makers among three Treg subsets. Autocrine IFN-*γ* was reported to regulate TGF-*β*-driven Foxp3 expression in induced regulatory T cells (iTreg) and suppress the conversion of naïve CD4 T cells into CD4^+^Foxp^+^ T cells [29]. We found lower IFN-*γ* in aTreg cells compared with non-Treg cells, suggesting that aTreg cells produce lower level of IFN-*γ* to suppress the antitumor immune responses. TGF-*β* serves as a pleiotropic regulator of essential functions in immune cells [30]. TGF-*β* signaling pathway can inhibit Tregs proliferation in thymus and promote CD8^+^ T cell maturation as well as NKT cell development [31]. But TGF-*β* is also required for Tregs development and survival. And no response to TGF-*β* can decrease the number of Tregs [31–33]. In this study, we found higher expression of TGF-*β* in aTreg cells. Among three subsets of Treg cells, aTreg which performed high proliferation, corresponding to HLA-DR-expressing and suppressing the proliferation of responder cells, was the main functional population of Tregs. It has been showed that the TGF-*β* pathway related genes have dysregulation between different Treg cell subsets [34], indicating that the high expression of TGF-*β* in aTreg cells was important for the Treg cells function. The high expression of TGF-*β* can induce a series of molecular events regulation which contributed to cell cycle, apoptosis, and others. Our findings supported the essential role of TGF-*β* for Tregs development and differentiation. CD39 can catalyze the conversion of extracellular ATP or ADP to AMP and act as another key mechanism of Tregs in suppressing antitumor immune response [35]. CD39 expressed on Tregs was reported to inhibit NK activity and promote hepatic metastatic tumor growth [36]. The increased expression of CD39 in CD4^+^ T cells was related to poorer prognosis [37]. We also found high expression of CD39 in aTreg cells from NCSLS patients. Polyoxometalate-1, an inhibitor of nucleoside triphosphate diphosphohydrolase activity, can effectively inhibit the Treg cells activity* in vitro* and the tumor growth* in vivo *[36]. Therefore inhibition of CD39 may promote the antitumor immune responses by suppression of Tregs and act as component of immunotherapy for cancer.

Lung cancer is the most common cause of cancer-related mortality worldwide, with 85% being of the NSCLC histological subtype [38]. Since we found the association of aTregs and the clinical stage of NSCLC, next we detected the portion of Tregs in NSCLC patients after chemotherapies. We found Treg cells decreased after treatment with docetaxel* in vitro*, but only aTreg cells decreased with more IFN-*γ* and less TGF-*β*. We also found that the three subsets of Treg cells were greatly reduced after 4 cycles of chemotherapy. This study of NSCLC patients agreed with our findings* in vitro*. Although the portion of aTregs was extremely lower after 4 cycles of chemotherapy, there are still aTreg and rTregs remaining in peripheral blood. It is already known that rTreg cells can differentiate to aTreg cells after stimulation if aTreg cells die. We considered this may contribute to the recurrence and poor survivals of NSCLC. The depletion of Treg cells can significantly prolong survival in combination with chemotherapy in preliminary studies; on the other hand, chemotherapy can decrease Treg cells to improve antitumor immunity [26, 39]. Our results combined with others shed light on the development of new therapeutic schedules combining chemotherapy with immunotherapy.

## 5. Conclusion

In conclusion, we found that aTreg cells significantly increased in NSCLC patients and associated with late clinical stages. In this study, we also found aTreg significantly decreased after effective chemotherapy. In molecular events, it showed that aTreg cells showed lower levels of IFN-*γ* and higher level of TGF-*β* and CD39. The chemotherapy drug docetaxel can decrease aTreg cells and change the expression of IFN-*γ* and TGF-*β*. This study indicates that the inhibition of TGF-*β* and CD39 to suppress Tregs may act as a component of immunotherapy for cancer. It also provides the potential of combination with chemotherapy and immunotherapy in the future.

## Figures and Tables

**Table 1:** Clinical and pathologic characteristics of patients (*n* = 40).

Characteristics	Number of patients	Proportion (%)
Sex	40	
Male	24	60
Female	16	40
Age (years)		
<60	10	25
≥60	30	75
Pathology		
Adenocarcinoma	20	50
Squamous	20	50
Stage		
I-II	8	20
III-IV	32	80

## References

[1] Zou W (2006). Regulatory T cells, tumour immunity and immunotherapy. *Nature Reviews Immunology*.

[2] Curiel TJ, Coukos G, Zou L (2004). Specific recruitment of regulatory T cells in ovarian carcinoma fosters immune privilege and predicts reduced survival. *Nature Medicine*.

[3] Stoop JN, van der Molen RG, Baan CC (2005). Regulatory T cells contribute to the impaired immune response in patients with chronic hepatitis B virus infection. *Hepatology*.

[4] Kriegel MA, Lohmann T, Gabler C, Blank N, Kalden JR, Lorenz H-M (2004). Defective suppressor function of human CD4^+^ CD25^+^ regulatory T cells in autoimmune polyglandular syndrome type II. *The Journal of Experimental Medicine*.

[5] Deaglio S, Dwyer KM, Gao W (2007). Adenosine generation catalyzed by CD39 and CD73 expressed on regulatory T cells mediates immune suppression. *The Journal of Experimental Medicine*.

[6] Mandapathil M, Szczepanski MJ, Szajnik M (2009). Increased ectonucleotidase expression and activity in regulatory T cells of patients with head and neck cancer. *Clinical Cancer Research*.

[7] Nadkarni S, Mauri C, Ehrenstein MR (2007). Anti-TNF-*α* therapy induces a distinct regulatory T cell population in patients with rheumatoid arthritis via TGF-*β*. *The Journal of Experimental Medicine*.

[8] Rao PE, Petrone AL, Ponath PD (2005). Differentiation and expansion of T cells with regulatory function from human peripheral lymphocytes by stimulation in the presence of TGF-*β*. *The Journal of Immunology*.

[9] Ito T, Hanabuchi S, Wang Y-H (2008). Two functional subsets of FOXP3^+^ regulatory T cells in human thymus and periphery. *Immunity*.

[10] Fontenot JD, Gavin MA, Rudensky AY (2003). Foxp3 programs the development and function of CD4^+^CD25^+^ regulatory T cells. *Nature Immunology*.

[11] Tran DQ, Ramsey H, Shevach EM (2007). Induction of FOXP3 expression in naive human CD4^+^FOXP3^−^ T cells by T-cell receptor stimulation is transforming growth factor-*β*—dependent but does not confer a regulatory phenotype. *Blood*.

[12] Sakaguchi S (2005). Naturally arising Foxp3-expressing CD25^+^ CD4^+^ regulatory T cells in immunological tolerance to self and non-self. *Nature Immunology*.

[13] Miyara M, Yoshioka Y, Kitoh A (2009). Functional delineation and differentiation dynamics of human CD4^+^ T cells expressing the FoxP3 transcription factor. *Immunity*.

[14] Lin Y-C, Mahalingam J, Chiang J-M (2013). Activated but not resting regulatory T cells accumulated in tumor microenvironment and correlated with tumor progression in patients with colorectal cancer. *International Journal of Cancer*.

[15] Mathian A, Parizot C, Dorgham K (2012). Activated and resting regulatory T cell exhaustion concurs with high levels of interleukin-22 expression in systemic sclerosis lesions. *Annals of the Rheumatic Diseases*.

[16] Curiel TJ (2007). Tregs and rethinking cancer immunotherapy. *The Journal of Clinical Investigation*.

[17] Bhattacharya K, Chandra S, Mandal C (2014). Critical stoichiometric ratio of CD4^+^ CD25^+^ FoxP3 ^+^ regulatory T cells and CD4^+^ CD25^−^ responder T cells influence immunosuppression in patients with B-cell acute lymphoblastic leukaemia. *Immunology*.

[18] Woo EY, Chu CS, Goletz TJ (2001). Regulatory CD4^+^CD25^+^ T cells in tumors from patients with early-stage non-small cell lung cancer and late-stage ovarian cancer. *Cancer Research*.

[19] Marshall NA, Christie LE, Munro LR (2004). Immunosuppressive regulatory T cells are abundant in the reactive lymphocytes of Hodgkin lymphoma. *Blood*.

[20] Ichihara F, Kono K, Takahashi A, Kawaida H, Sugai H, Fujii H (2003). Increased populations of regulatory T cells in peripheral blood and tumor-infiltrating lymphocytes in patients with gastric and esophageal cancers. *Clinical Cancer Research*.

[21] Chan OTM, Yang L-X (2000). The immunological effects of taxanes. *Cancer Immunology, Immunotherapy*.

[22] Schuler PJ, Schilling B, Harasymczuk M (2012). Phenotypic and functional characteristics of CD4^+^ CD39^+^ FOXP3^+^ and CD4^+^ CD39^+^ FOXP3^neg^ T-cell subsets in cancer patients. *European Journal of Immunology*.

[23] Mandapathil M, Hilldorfer B, Szczepanski MJ (2010). Generation and accumulation of immunosuppressive adenosine by human CD4^+^CD25^high^FOXP3^+^ regulatory T Cells. *The Journal of Biological Chemistry*.

[24] Turk MJ, Guevara-Patiño JA, Rizzuto GA, Engelhorn ME, Houghton AN (2004). Concomitant tumor immunity to a poorly immunogenic melanoma is prevented by regulatory T cells. *The Journal of Experimental Medicine*.

[25] Beyer M, Schultze JL (2006). Regulatory T cells in cancer. *Blood*.

[26] Ganesan A-P, Johansson M, Ruffell B (2013). Tumor-infiltrating regulatory T cells inhibit endogenous cytotoxic T cell responses to lung adenocarcinoma. *The Journal of Immunology*.

[27] Yu P, Lee Y, Liu W (2005). Intratumor depletion of CD4^+^ cells unmasks tumor immunogenicity leading to the rejection of late-stage tumors. *The Journal of Experimental Medicine*.

[28] Borsellino G, Kleinewietfeld M, di Mitri D (2007). Expression of ectonucleotidase CD39 by Foxp3^+^ Treg cells: hydrolysis of extracellular ATP and immune suppression. *Blood*.

[29] Chang J-H, Kang C-Y (2008). Autocrine IFN-gamma directly regulates Foxp3 expression in naive CD4^+^CD25^−^ T cells. *The FASEB Journal*.

[30] Letterio JJ, Roberts AB (1998). Regulation of immune responses by TGF-*β*. *Annual Review of Immunology*.

[31] Li MO, Sanjabi S, Flavell R (2006). Transforming growth factor-*β* controls development, homeostasis, and tolerance of T cells by regulatory T cell-dependent and -independent mechanisms. *Immunity*.

[32] Sledzinska A, Hemmers S, Mair F (2013). TGF-beta signalling is required for CD4^+^ T cell homeostasis but dispensable for regulatory T cell function. *PLOS Biology*.

[33] Sojka DK, Fowell DJ (2011). Regulatory T cells inhibit acute IFN-*γ* synthesis without blocking T-helper cell type 1 (Th1) differentiation via a compartmentalized requirement for IL-10. *Proceedings of the National Academy of Sciences of the United States of America*.

[34] G E, Haribhai D, Williams JB (2012). IL-10 produced by induced regulatory T cells (iTregs) controls colitis and pathogenic ex-iTregs during immunotherapy. *The Journal of Immunology*.

[35] Parodi A, Battaglia F, Kalli F (2013). CD39 is highly involved in mediating the suppression activity of tumor-infiltrating CD8^+^ T regulatory lymphocytes. *Cancer Immunology, Immunotherapy*.

[36] Sun X, Wu Y, Gao W (2010). CD39/ENTPD1 expression by CD4^+^Foxp3^+^ regulatory T cells promotes hepatic metastatic tumor growth in mice. *Gastroenterology*.

[37] Perry C, Hazan-Halevy I, Kay S (2012). Increased CD39 expression on CD4^+^ T lymphocytes has clinical and prognostic significance in chronic lymphocytic leukemia. *Annals of Hematology*.

[38] Jemal A, Bray F, Center MM, Ferlay J, Ward E, Forman D (2011). Global cancer statistics. *CA: A Cancer Journal for Clinicians*.

[39] McCoy MJ, Lake RA, van der Most RG, Dick IM, Nowak AK (2012). Post-chemotherapy T-cell recovery is a marker of improved survival in patients with advanced thoracic malignancies. *British Journal of Cancer*.

